# Structural stressors, neurocognition in reward-related decision making and substance use risk in Puerto Rican early adolescents at two sites: study design

**DOI:** 10.3389/fpubh.2026.1663799

**Published:** 2026-05-19

**Authors:** Tamara J. Sussman, Ligia M. Chavez, Melanie Wall, Frances R. Levin, Lillian Polanco-Roman, Tenneill M. Murray, Cristiane S. Duarte

**Affiliations:** 1 Psychiatry Department, Columbia University, New York, NY, United States; 2 Universidad de Puerto Rico, Medical Sciences Campus, Behavioral Sciences Research, San Juan, Puerto Rico; 3 New York State Psychiatric Institute, New York, NY, United States; 4 Psychology Department, New York University, New York, NY, United States

**Keywords:** adolescent substance use risk, structural stressors, reward-related decision making, delay discounting, MRI, Puerto Rican adolescents

## Abstract

**Background:**

Structural stressors (SS) or mutually reinforcing systems that prevent groups of individuals from accessing opportunities and resources, can impact one's health. Indicators of SS include area-level group differences in income, education and exposure to violence. SS are fundamental causes of health disparities and can be indexed by area-level measures. Area-level SS may influence substance use risk in adolescents and future substance use disorders (SUDs). SUDs often start during adolescence, a critical risk period and an ideal time for prevention and intervention efforts. Since SUD risk can be influenced by the experience of the prior generation, it is important to take into account familial risk when trying to understand the role of SS on substance use risk. Improved understanding of the relationship between SS and neurocognitive mechanisms of substance use risk could inform novel approaches to prevention and intervention.

**Methods:**

We will enroll participants who are part of the Boricua Youth Study (BYS), an intergenerational, representatively sampled cohort of Puerto Rican families living in the South Bronx, New York and San Juan, Puerto Rico that has been followed for 20+ years. Working with BYS will allow us to control for familial SUD risk with prospectively captured parental SUD data and examine the how SS is related to substance use risk in two disadvantaged contexts. This article describes a pilot study focused on providing proof of the concept that specific area-level indicators of SS, including measures of income, education and exposure to violence, relate to neurocognitive mechanisms of substance use risk, including steeper delay discounting and reduced reward-related neural activity in reward-sensitive brain regions. Analyses will test a relationship between SS and neurocognitive mechanisms of substance use risk.

**Discussion:**

Results will reflect associations between area-level, substance use-relevant SS and neurocognitive mechanisms of substance use risk in adolescents. If proof of concept is obtained, the next phase of the study will examine structural and family-level factors that may buffer the negative impact of SS. The goal of this research is to guide novel approaches to structural-, family- and individual-level prevention of SUD.

**Clinical trial registration:**

https://clinicaltrials.gov/study/NCT06221839, identifier: NCT06221839.

## Background

Adolescence is a critical risk period for the initiation of substance use, characterized by significant neurodevelopment and social changes (1–4). Substance use disorders (SUDs) often start with adolescent substance use (5), which is common in the US. For example, in 2023, the prevalence of past month alcohol use was 15% by 8th graders, 31% by 10th graders and 46% by 12th graders, and the prevalence of last year cannabis use was 8% by 8th grades, 18% of 10th graders and 29% of 12th graders (6). Rates of SUD are lower than rates of adolescent substance use; for example, 2.4% of 14–15 year olds and 5.7% of 16–17% met criteria for alcohol use disorder and 5.3% of 14–15 year olds and 9.0% of 16–17 year olds met criteria for cannabis use disorder (7). In young adulthood, SUD prevalence rates are higher, with 27.1% of 18–25-year-olds meeting the criteria for a SUD in the previous year (8). Young adults with a SUD often do not get the treatment they need. For example, among young adults, aged 18–25, while 28.7% need SUD treatment – e.g., either have a current SUD diagnosis or have received treatment for SUD in the last year – only 4.7% reported receiving treatment (8). The large percentage of young adults with untreated SUD can create long-term burdens for individuals, their families, and society. Since substances alter brain development (9), it is ideal to examine the relationship between structural stressors and neural correlates of substance use risk before substance use initiation. Therefore, the current study will recruit participants who are 11–14 years old, with the expectation that the majority of participants will not have initiated substances use. While early use increases risk for SUD, many adolescents explore substance use, but do not progress to SUD (10). Thus, improved understanding of the neurocognitive mechanisms of substance use risk in early adolescence could elucidate both risk and protective factors, with the long-term goal of developing novel approaches to prevention.

SUDs are defined in the DSM-5 TR as a pathological pattern of behaviors related to substance use including impaired control over substance use, social impairment, risky use of substances, and pharmacological criteria, and are operationalized as meeting at least two out of 11 substance-specific criteria within a specific time frame, e.g., the past 12 months (11). SUD rates vary by demographic group and substance. For example, in 2023, past year rates of SUD were highest for Indigenous Americans (25.3%), and Multiracial Americans (24.3%), compared to White (17.8%), Latine (15.7%) and Asian Americans (9.2%) (8). However, examining SUD rates in broad demographic groups, such as “Latine” or “Indigenous Americans” can obscure meaningful differences between subgroups. For example, while recent US prevalence rates for SUD among Latine individuals may be comparable with total US rates (8) significant differences in adult SUD rates between Latine subgroups have been observed, with higher lifetime rates of SUDs and annual incidence of alcohol use disorder in Puerto Rican adults than other Latine subgroups (12, 13). Studies examining substance use have found similar results. For example, a US study examining alcohol consumption in Latine subgroups found that adults of Puerto Rican origin was a significant predictor of weekly alcohol consumption and of binge drinking (β = 0.50), while controlling for socioeconomic factors, including educational attainment, household income and employment status (14). Thus, Puerto Rican adolescents may be at particularly high risk for substance use and SUDs, and better understanding substance use risk in early adolescence in this group will provide actionable results that shape more effective preventions and interventions.

Familial risk is a known predictor of substance use (15–17), and significantly higher rates of SUD are reported in prospective compared to retrospective report (18). Therefore, to isolate the impact of SS on substance use risk, it is crucial to control for prospectively captured familial SUD risk data. In addition, parental risk factors, such as parental substance use, parental levels of sensation seeking, and impulsivity may also influence their children's substance use risk. A strength of the current study is that we are able to address these issues by collecting data from the Boricua Youth Study (BYS; more fully described below), such that at least one parent of each child we will recruit will have provided clinical information at up to 4 waves of the BYS study, providing data regarding parental SUD diagnosis, level of substance use, sensation seeking and impulsivity.

Structural stressors (SS), defined as mutually reinforcing systems that place individuals from some groups at a disadvantage, are recognized as a fundamental cause of disparities in health (19). For example, SS, including the poverty gap between minoritized and majority groups, have been related to lower levels of wellbeing in children (20–22). Systems of income, education, and housing opportunities can reinforce each other such that some individuals receive fewer opportunities, e.g., parents who earn less money may live in school districts where their children receive lower quality education, making it harder to reach other educational and employment opportunities (19, 23). In addition, these mutually reinforcing systems create environments in which individuals face higher exposure to adversities, leading to more negative health outcomes (19, 24–26). Commonly, but not without limitations, area level measures such as neighborhood income or economic opportunity are used to index SS (20–22).

Stressors seem to play an important role in SUD-related outcomes. For example, exposure to community violence is associated with increased odds of substance use in youth (27), and economic downturns relate to increases in clinically relevant substance use disorders involving opioids (28). Similarly, youth belonging to disadvantaged groups, including ethnoracially minoritized youth, have less access to treatment for SUDs than their White counterparts (29). SS relate to increased substance use in Black adults (30) and to county-level overdose deaths (31). However, the role of SS on substance use in adolescents at higher risk for SUDs is not known. This represents a major gap considering that constructs related to SS measured during adolescence e.g., experiences of unfair treatment, i.e., routine, daily experiences of interpersonal mistreatment, such as being treated with less respect, courtesy or fairness than others due to being part of specific racial or ethnic group, low socioeconomic status, low educational attainment, or being exposed to violence are all associated with higher prevalence of substance use (32–40).

Puerto Rico is a US Commonwealth, which provides a unique set of rights and burdens to Puerto Rican individuals. For example, Puerto Rican individuals have US citizenship (41), which may be protective from some, but not all, harms that other Latine groups in the US may experience (42). However, Puerto Ricans individuals living on the island of Puerto Rico do not have voting representation in the US Senate or House of Representatives and are not entitled to electoral votes for presidential elections unless they are living in the continental US (41). Furthermore, policies impacting Puerto Rico place economic burdens on people living on the island (43). For example, the Jones Act, enacted in 1920 and still in effect (with minor gaps throughout the years), mandates that all goods within the coasts of the US (with a few exceptions) must be transported using U.S.-owned and -operated vessels (44). This slows the pace of trade, adds taxes to all shipped goods and disproportionally harms places like Puerto Rico that rely heavily on ocean shipping (43). Thus, the history and political landscape of Puerto Rico likely exert substantial structural influences and SS on Puerto Rican individuals and families and could potentially contribute to higher SUD rates in Puerto Rican adults than in other Latine groups in the US. Examining the role of SS on adolescent substance use risk in a group at higher risk for SUDs is a priority for better understanding the relationship between SS and neurocognitive mechanisms of substance use risk.

In addition to meaningful differences in between Latine subgroups, meaningful differences in SUDs have been found in between Puerto Rican young adults living on the island of Puerto Rico compared to those living on the continental US, such that past-year prevalence of illicit SUD was significantly higher on the continental US (7.8%) than on the island of Puerto Rico (3.7), χ^2^ = 11.1 (45). The two-site design of this pilot study will allow us to explore the relationship between SS and neurocognitive mechanisms of substance use risk in two distinct contexts: the continental US and the island of Puerto Rico. Thus, our preliminary results from our study could indicate if SS are relevant to substance use risk for Puerto Rican children who live on the island of Puerto Rico or on the continental US. The second, larger phase of the study will be better powered to examine site-related differences than the pilot phase.

Denied opportunities, such as lack of access to appropriate income, high quality educational settings, and a safe living environment, tend to cluster together geographically and to reinforce each other. Thus, high levels of SS in the US can lead to appraising the environment as unjust. Belief in a just world (BJW), or the belief that people generally receive what they deserve, has been theorized to develop during adolescence, to allow individuals to see the world as predictable and to allow individuals to pursue long-term goals (46). In addition, learning to wait for larger, later rewards has been theorized to emerge in adolescence, alongside BJW, as it would be sensible to wait for larger rewards in a world where people are rewarded or punished according to their behavior (46), and not due to factors beyond their control. Consistent with this theory, research examining the relationship between BJW and reward-related decision making has found that weaker BJW relates to choosing smaller, sooner over larger, later rewards, aka steeper delay discounting (DD) (47, 48).

Exposure to negative experiences have been shown to alter reward-related decision making (49–51). For example, among trauma exposed adults, trauma severity relates to decreased model-based learning related to rewards (49). Similarly, in a systematic review, exposure to adverse childhood experiences was associated with attenuated reward learning (50). Among studies that frame experience-related changes in task performance results as adaptation, rather than using a deficit-based approach, adverse childhood experiences were examined in relation to the trade-off between exploiting a known source of diminishing rewards compared to exploring a novel source with a fresh distribution of rewards (51). Researchers found that adults exposed to adverse childhood experiences tended to explore less (51). This is consistent with the previous studies finding that negative experiences relate to a lower learning rate and indicates that recent feedback about rewards may be less integrated into decision-making. Relying more on long-term trends in decision-making and downplaying the importance of recent events may be the best strategy in a volatile environment, such as one where adverse childhood experiences occur often. If exposure to high levels of SS tend to shape reward-related decisions across a group of individuals, this information could be used to help shape policy and structural factors to protect certain groups from the negative effects of SS.

Choosing the currently available reward is the optimal strategy in an unjust world in which long-term investments are not paid off. For example, in a variant of Mischel's marshmallow task, children were either placed in a “reliable” environment, in which rewards were given as promised, or an “unreliable” environment, in which children were told promised rewards were no longer available. Children in the reliable environment were willing to wait longer for the marshmallow reward than children in the unreliable environment (52). Consistent with these results, studies of adolescents have found economic status may drive strategic reward-related decision making. For example, lower SES is associated with attenuated behavioral and neural responses to rewards (53, 54), and less exploration on a reward learning task, a pattern of behavior that has been linked to academic performance in previous research (55). Furthermore, the impact of economic status during childhood has been shown to have a lasting effect on adults such that those with lower childhood SES have been found to be more risk averse, and more prosocial while engaging with economic games than adults with high SES (56). Finally, studies of deprivation to different kinds of rewards, e.g., fasting and social isolation, suggest that reward responsive brain regions contribute to craving in response to deprivation (44, 57), potentially identifying a neural mechanism explaining how greater exposure to SS could lead to changes in reward-related decision making. Thus, across a wide range of task-types, the availability of rewards in the environment seems to impact how children, adolescents and adults perform reward related decisions, with different paradigms finding a preference for more immediate rewards following experiences of deprivation.

Consistent with this research, in studies examining the experience of deprivation at the neighborhood level, children living in zip codes with a high Area Deprivation Index demonstrate blunted recruitment of reward-implicated brain areas during reward anticipation (58). Furthermore, children from lower-resourced areas, according to the Child Opportunity Index, were found to have reduced connectivity between the control and motor brain networks during a reward-related decision making task (59). However, it is currently unclear how SS, measured as differences among groups within a geographic area, indexing structural inequities at the area level, relate to reward related decision-making. Importantly, if, as hypothesized, area-level SS are relevant, interventions that simply improve area level social factors (e.g., income level), without changing structural social differences between groups (SS) are unlikely to improve outcomes.

Higher levels of BJW are associated with greater resilience and optimism in adolescents (60), however, the benefits of higher levels of BJW are not equally shared across all adolescents, with racially and socioeconomically privileged adolescents having higher BJW scores than adolescents from groups who are less privileged (61). Experimental results support a relationship between BJW and reward related decision making. Exposure to unjust scenarios, e.g., a video describing the suffering of an innocent victim, and lower self-reported BJW are both associated with steeper DD (47, 48). Furthermore, compared to a control condition, reading an article describing increasing fairness was related to increased willingness to invest in long term goals for participants who identified as belonging to an ethnic minority (62). In another study, the inverse association between perceived discrimination and subjective wellbeing was found to be completely mediated by BJW, suggesting that experiences of discrimination undermine BJW, thus leading to worse outcomes on self-reported wellbeing (63). While BJW has been related to individual life experiences, such as experiences of discrimination (63), the relationship between BJW and area-level SS, defined as stressors reflecting mutually reinforcing systems that place individuals from some groups at a disadvantage, has not been explored, to our knowledge. Growing up in an environment where area-level differences in SS are present, e.g., a census tract with more people in one group representing the top income quintile and another group overrepresented in the bottom income quintile, could lead to the perception of the world as less just, and thus could lead to the development of steeper DD.

However, steeper DD has also been associated with later alcohol use (64) and with SUD diagnosis (65–67). This is theorized to occur when a future outcome (e.g., being able to focus in class the next day, or avoiding a hangover) is devalued and a more immediate reward, (e.g., enjoying alcohol) is chosen (68). Thus, in the context of SUD research, steeper DD has been interpreted as a sign of maladaptive impulsivity, while waiting for larger, later rewards is assumed to be optimal behavior. While steeper DD may be an adaptive strategy in contexts where long-term investments do not pay off, such as areas characterized by high levels of SS, when the reward in question is a substance, and immediate use is chosen over long-term health, this adaptation to the environment could increase the odds of negative outcomes related to substance use (69).

Neuroscience research describing the neurodevelopment of children and adolescents has had a significant impact on policy. More specifically, studies demonstrating the effect of poverty on neural structure in childhood were cited in policy briefs and reports supporting child tax credits (70–73). Furthermore, research demonstrating the differences between adolescent and adult brains helped to form Supreme Court decisions protecting individuals who committed crimes during adolescence from punishments such as the death penalty and life sentence without the possibility of parole (74–77). Thus, characterizing the neurocognitive effects of SS on the adolescent brain may provide important support for advocacy for structural interventions, the most direct solution to structural disparities.

Neighborhood stressors have been shown to relate to brain volume in children. For example, neighborhood level poverty/deprivation is associated with reduced brain volume in children. Several studies have replicated a relationship between neighborhood disadvantage or opportunity and total brain volume, such that greater advantage or lower levels of opportunity relate to reduced total brain volume (78–81). In addition, other studies have found a more specific effects of area-level stressors, including greater neighborhood disadvantage and lower childhood opportunities on prefrontal brain regions (82) including superior frontal gyrus, dorsal lateral and medial prefrontal cortices (78, 79, 83). These previous studies have been conducted in younger children, however, adolescence is characterized by significant maturation in prefrontal regions (84, 85). Changes in neural structure related to neighborhood stressors could indicate neural shaping by the environment (78), possibly to prepare individuals for later life in a similar environment. Neural structure is theorized to provide the conditions for neural function without determining the neural network dynamic (86). Remodeling of structure–function coupling has been documented during adolescence, with, for example, coupling in prefrontal regions relating to executive function (87). Therefore, if we find a relationship between SS, measured as area-level differences among groups, and brain structure, this could index environmental shaping of the brain, and could relate to subsequent neural function. Thus, examining the relationship between SS and prefrontal volumes during adolescence is a novel and important next step to understanding how SS influence neurodevelopment and cognitive development.

Given the relevance of DD in reward-related decision making and in SUDs, the neural correlates of DD have been examined via meta-analysis. Activity in the dorsal medial prefrontal cortex (dmPFC) and anterior cingulate cortex (ACC) have been associated with reward valuation, while decreased activity in inferior frontal gyrus (IFG/insula) is associated with a stronger preference for immediate compared to delayed rewards (88). Furthermore, activity in the prefrontal and insular regions are key nodes of a brain marker of individual differences in DD task performance, demonstrating their importance for cognitive processes related to this task (89). The ventral striatum (VS) is a reward-sensitive brain region, activity in the VS increases both when a reward is anticipated and when it is received, and previous studies have linked DD-related VS activity with reward value in adolescents (90). Individual differences in DD correlate positively with magnitude of VS activation such that steeper DD was associated with increased activity in VS (91). Individual differences in delay discounting relate to patterns of connectivity seeded in the dorsal prefrontal cortex (92). Ventral striatal connectivity mediates the relationship between DD performance and subsequent negative health outcomes, including increased BMI in adolescents (93), results underlining the centrality of the dorsal PFC and ventral striatum to DD performance. These results from the existing literature suggest that lower task-related activity across regions including dmPFC, ACC, IFG/insula, and VS may be a risk factor for substance use. Experiences of discrimination and social exclusion have been associated with distinct alterations in PFC functioning, and the ACC is likely a key region for processing social exclusion (94, 95). Thus, the dmPFC, ACC, IFG/insula and VS emerge as key brain regions of interest given their association with both substance use and social exclusion.

Three distinct dual systems models describe diverging growth curves for the neurobiological systems underlying reward sensitivity and cognitive control across adolescence in into adulthood. The Dual Systems Model (96), the Maturational Imbalance Model (97), and the Driven Dual Systems Model (98) all propose that during early adolescence, the reward-sensitive VS develops relatively quickly compared to the slower development of prefrontal regions associated with cognitive control, and this mismatch in development is theorized to explain why adolescence is life stage characterized by increased in risky behaviors, including substance use (98–105). The three dual systems models differ in their hypotheses regarding the growth curves of reward sensitivity and cognitive control during later adolescence and early adulthood, suggesting different outcomes during these later life stages (96–98). However, all three models agree that studying neural activity in reward-sensitive brain regions and prefrontal regions associated with cognitive control can contribute to our understanding of adolescent reward-related decision-making (100). For all these reasons, the dmPFC, ACC, IFG/insula and VS regions are particularly important to study, at this developmental stage, in relation to substance use risk (98, 100, 102–105).

*The Boricua Youth Study: An opportunity to isolate the role of SS on substance use, by controlling for familial factors*.

The Boricua Youth Study (BYS) is an epidemiological study formed with the goal of better understanding the lived experiences of Puerto Rican individuals and their families living in two contexts: the island of Puerto Rico, and the New York City region of the continental US (106). BYS started in 2001 through a collaborative effort between NYSPI/Columbia University, led by Hector Bird, and the University of Puerto Rico, led by Glorisa Canino. BYS originally recruited about 2,500 children and their families based on multistage probability household samples, representative of the two original target populations: Puerto Rican families in the South Bronx, NY and in the metropolitan area of San Juan, Puerto Rico. The original cohort of BYS children were aged 5–13 at the first wave of recruitment and data were collected over 3 Waves, between 2001–2004 (106). A fourth assessment wave, collected during young adulthood, was collected between 2013–2018 (107, 108).

By leveraging an existing intergenerational, longitudinal study with representative samples of Puerto Rican families in two contexts, this study will be the first to examine the relationship between SS and neurocognitive mechanisms of substance use risk, while controlling for familial risk for SUD. Results will help inform larger future studies, with the long-term goal of (1) shaping policy targeted at reducing SS-related health risks and (2) suggesting novel family- and individual-level mechanisms that can inform approaches to prevention and intervention.

## Methods

This study was funded as an R61 pilot award and aimed to establish proof of the concept that SS, measured as area-level differences among groups, is associated with neurocognitive mechanisms of substance use risk. R61 studies are awarded to conduct exploratory research that will provide evidence for a second phase of research. The goal of this R61 is to provide initial results to support funding for a larger study with the goal of replicating the association between SS and neurocognitive mechanisms of substance use risk, additionally testing the relationship between area-level SS and substance use risk, substance use, and examining the role of protective factors at the structural and family level.

### Participants

We will invite 72 children from the second generation of the BYS, aged 11–14 years old, 50% female, 50% from the South Bronx cohort, 50% from the San Juan cohort, and at least 1 parent, to participate in the proposed pilot study by providing residential history (parental report) and belief in a just world (child report) via interview, delay discounting task performance and neural correlates via an MRI scan.

**R61 Specific Aims, Proof of Concept of a relationship between SS and Neurocognitive Mechanisms of Substance Use:**

**Figure 1:**
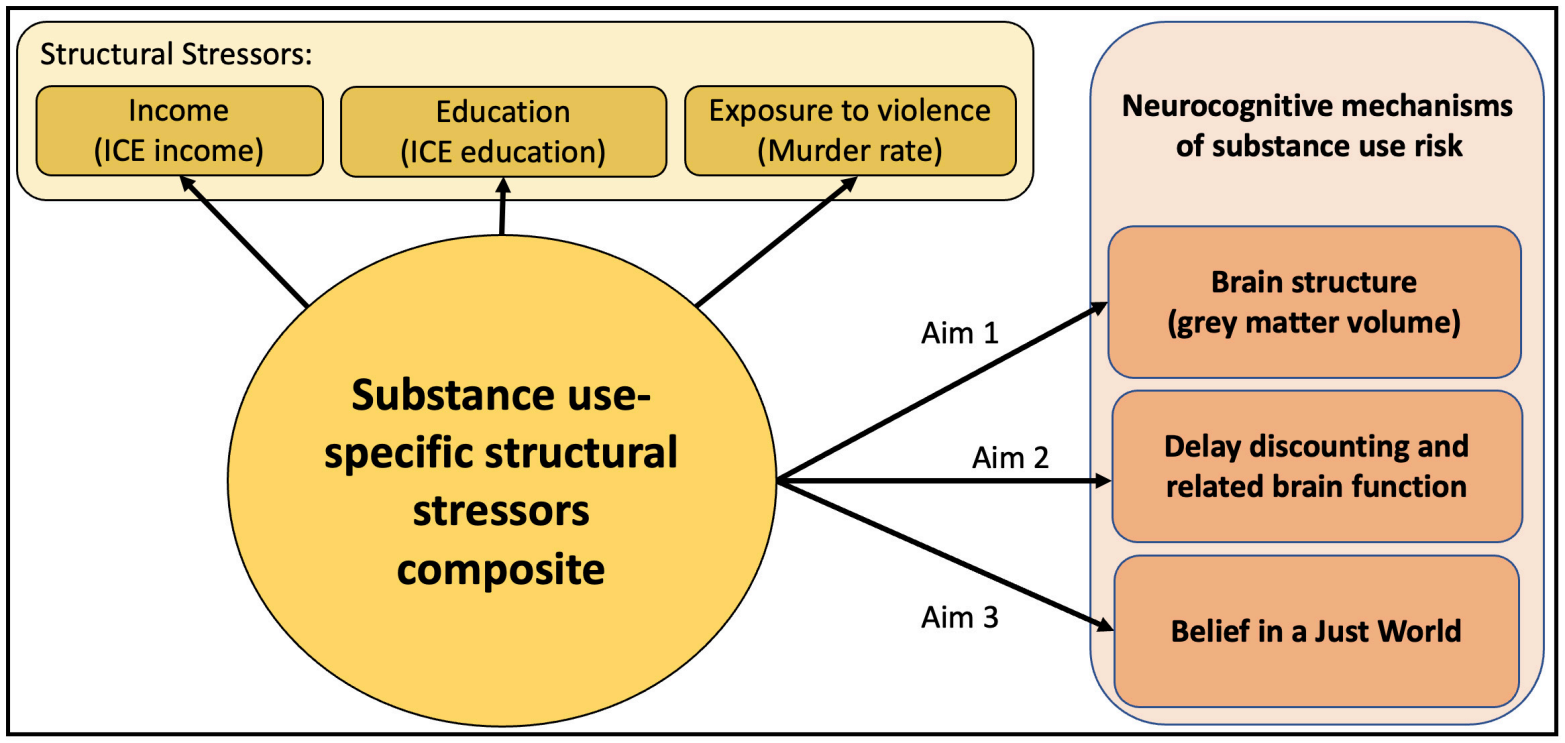
R61 phase aims. Hypotheses: A substance-use specific composite of structural stressors will relate to brain structure, steeper delay discounting (DD), DD–related brain function, and belief in a just world. DD is theorized to develop alongside belief in a just world, as it is sensible to wait for larger rewards in a just world, where people get what they deserve. Steeper DD may be an adaptive response to an environment high in structural stressors. However, steeper DD is also an established correlate of SUD, and thus, this adaptive response to the environment could increase substance use risk.

**Aim 1, Hypothesis 1, SS & brain structure:** Higher levels of SS, as measured by our substance use-relevant SS composite, will relate to adolescent brain structure, Figure 1 including lower volume of superior frontal gyrus, dorsal lateral and medial prefrontal cortices.

**Aim 2, Hypothesis 2, SS & DD:** We expect that greater SS will relate to steeper DD, and less DD-related brain activity in dorsal medial prefrontal cortex, anterior cingulate cortex, insula and ventral striatum. We will use the SS composite variable as a predictor and task-related brain activity in regions of interest (ROIs) as the outcome.

**Aim 3, Hypothesis 3, SS & BJW:** Greater SS composite will relate to weaker BJW.

A moderate effect size association (r > 0.30) found for Hypothesis 1, Hypothesis 2, or Hypothesis 3 will justify progression to the R33 phase.

### Composite of SS

Because SS are mutually reinforcing systems (19), we plan to create a substance use-relevant SS composite to represent how these SS co-occur. To select a set of specific stressors to create a substance use-relevant SS composite, existing empirical data supporting the relevance of individual- or area-level social stressors for substance use, when possible, in adolescence was examined. Specifically, lower childhood socioeconomic status is associated with increased risk for substance use disorders (109, 110). Adolescents from low-income families living in high income neighborhoods were at greater risk for smoking compared to adolescents from low-income families living in low income neighborhoods, and compared to adolescents from high-income families (111). Youth who drop out of high school are more likely to engage in substance use or misuse and to meet criteria for substance dependence compared to their peers who stay in high school (37, 112, 113). Increased perception of neighborhood violence and exposure to violence is associated with increased substance use (38, 114). Other social factors considered for the SS composite were less consistently associated with substance use risk. For example, while residential segregation has been consistently associated with differential access to substance use treatment (115, 116), the relationship between segregation and substance use risk is less clear (117, 118). Similarly associations with access to green spaces and substance use risk have been found to have a less consistent relationship with substance use (119). Therefore, generating a composite of SS using census tract level group differences in income, educational attainment, and precinct level murder rates as indices will provide a measure of SS that is relevant for adolescent substance use risk.

### Measurement of SS

**Figure 2:**
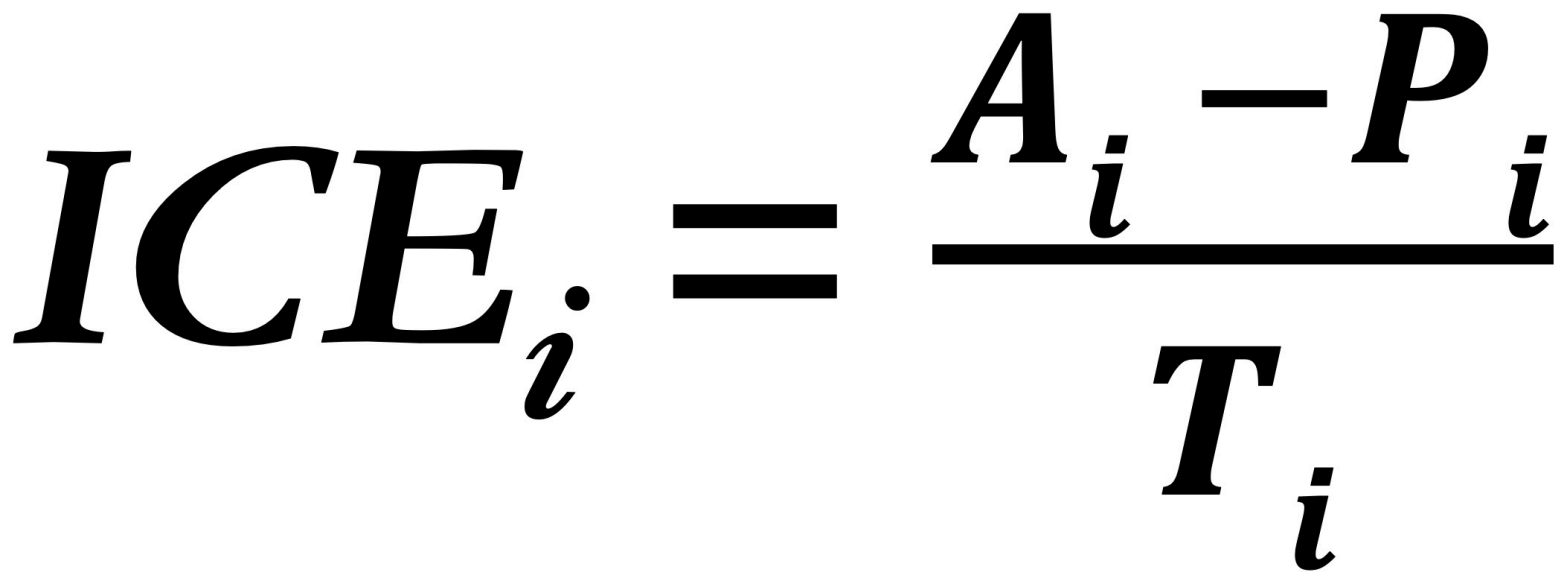
The ICE formula, from Krieger et al. (22).

To determine the role of SS on substance use risk in adolescents, we will create a composite measure of substance use-relevant SS. This composite will be based on area-level racial group differences in income, education, and exposure to violence, as exposure to lower levels of income and education, and higher levels of violence have been related to increased risk for substance use (2–9). To calculate SS SU-relevant measures of income and education, we will use the Index of the Concentration at the Extremes using income and education, using data from the US census. For example, for income, within each census tract (i) our participants live in, the ICE_income_ will be calculated as the number of White individuals in the top income quintile (Ai) minus the number of racially minoritized individuals in the bottom income quintile (Pi), divided by the total population across all income quintiles (Ti; Figure 2).

Previous studies report a relationship between ICE_income_ and a wide array of health disparities including infant mortality, child mortality, premature mortality, and exposure to black carbon, and it has been effectively used in small geographical units, including at the census tract level (21, 22, 120). ICE_education_ will be calculated within each census tract (i) our participants live in as the number of individuals 25 years or older (Ai) with 4 years of college or more who are White, minus the number of individuals who did not finish high school who are racially minoritized, divided by the total population across all education levels.

Previous analyses of socioeconomic factors in our BYS sample have demonstrated variation in both within and across sites, supporting our ability to examine the research questions described here. For example, within the continental US BYS sample, 56.7% of participants at the young adult assessment reported meeting the federal poverty threshold during young adulthood, while the remaining 43.3% reported not meeting this threshold (108). In the continental US 59.3% of the mothers of our participants had obtained a high school diploma or GED, again demonstrating variation among our sample. In PR, 65.0% of participants met the poverty threshold, and 80.8% of the mothers of our participants had done so in PR (108), demonstrating variation within the PR sample, and across the two sites of the BYS.

Disparities in exposure to violence will be measured using differences between the number of murdered White individuals and the number of murdered minoritized individuals at the precinct-level, divided by the total precinct population. In the continental US, these data can be obtained via requests to the National Crime Victimization Survey (NCVS), the primary source of national data on criminal victimization, at the precinct level. Crime data for PR is reported through the Uniform Crime Report (UCR) Program through the Government of Puerto Rico. This measure is distinct from measures that capture, for example, individual direct experiences of violence, or area-level measures that reflect the presence of violence in a certain neighborhood, rather, the proposed measure captures the extent to which, at the area-level, one group is exposed to violence compared another group. Using a composite of these SS indicators, we aim to generate preliminary support for the hypothesis that SS will relate to neurocognitive mechanisms of substance use risk.

### Participant assessments

Interviews of parent and child participants, and MRI scans of children will collect data using the following methods:

#### Assessment of belief in a just world

Learning to wait for larger, later rewards is theorized to develop alongside, and in conjunction with a BJW (65). We will use the 13 item self-report Belief in a Just World scale, which uses a Likert-type scale, and has been previously used with adolescent populations (121, 122). This scale, as a whole, has a Cronbach's alpha of 0.827, which is considered good internal consistency, indicating high reliability (121).

#### Assessment of collection of MRI data

Children will be introduced to scanning protocols in a mock scanner session and encouraged to finish participation through frequent praise and reminders to respond to the task, and to stay awake during the resting state scan. Multimodal MRI scanning will include a structural MRI (~5 min) and DD task (~10 min).

#### Assessment of DD behavior and neural correlates

We will use an fMRI-adapted DD task previously used with adolescents to collect a DD performance and related brain activity. This task has good test-retest reliability for behavior and neural response (123). Like other monetary DD tasks, on any given trial, participants are asked to decide between a smaller sooner reward, or a larger later reward. Participants will first perform a calibration task comprised of 50 trials to assess each individual child's discount parameter *k* outside of the scanner. This discount parameter is used to create 90 trials presented in the scanner, in which the participant can be expected to choose the smaller, sooner reward half of the time, providing adequate data to examine neural response to smaller soon and larger later rewards. Participants are told that they will be paid the amount of one of their choices after the task is completed.

#### Scanning parameters

At both sites, we plan to collect data using a 3T MRI scanner, using a 64-channel coil. For structural scans, a T1-weighted magnetization-prepared rapid acquisition with gradient echo (MPRAGE) sequence will be collected (TR = 1,900 ms, TE = 2.26 ms, FOV = 256 × 256 mm, 176 slices, 1 × 1 × 1 mm^3^ voxel size, flip angle = 9°). For functional, task-based imaging, a gradient-echo T2^*^-weighted EPI sequence (TR = 2,000 ms, TE = 25 ms, 2 × 2 × 2 mm^3^ voxel size, xflip angle = 80°) will be collected.

#### Assessment of covariates: familial SUD risk factors

To control for familial risk, we will use data collected about one of the parents of the participant during Waves 1–3 of the BYS. More specifically, we will account for (1) parental sensation seeking during childhood/adolescence (depending on the age of the parent when they participated in BYS), captured by the Sensation Seeking Scale for Children (124); (2) parental impulsivity symptoms during childhood/adolescence (ADHD via DISC-IV) (125); (3) substance use and SUD status during childhood/adolescence (DISC-IV) (125); and (4) parental SUD during young adulthood, as measured by the CIDI (126).

#### Assessment of covariates: unfair treatment

This study is focused on the role of SS, which can operate outside of an individual's perception of unfair treatment. Therefore, to isolate the role of SS from the role the perception of unfair treatment due to race or ethnicity, we will control for experiences of ethnoracial discrimination using the Perceptions of Racism in Children and Youth Scale (PRaCY), a 10-item child self-report scale (127). The PRaCY has strong internal consistency reliability, and confirmatory factor analysis of the scale demonstrates good fit.

#### Assessment of additional covariates

Other covariates of interest also include age, sex, and psychopathology (DISC-IV, for consistency with assessment of the parental generation (125), and the CBCL/6-18) (128). Since ADHD medication are prescribed to more children in the US than any other class of medication (129), and since ADHD medication has been shown to impact neural activity (130), analyses of neural data will take into account ADHD medication, which is reported on the DISC (125).

#### Procedures

The proposed pilot study will not be advertised or publicized, rather, we will reach out to families who have participated in BYS to determine eligibility for the current study.

#### Power analysis

Since R61 grants are awarded to conduct exploratory research focused on establishing a proof of concept, the power analysis for this R61 will focus on effect size rather than statistical significance of associations. Previous research has examined the effect sizes related to associations between stressors and delay discounting behaviors. For example, meta-analysis of studies examining the relationship between individual experiences of stress, e.g. adverse life events, perceived stress, or allostatic load, and delay discounting found moderate to large effects (131). We are aware of only two previous published studies examining the relationship between an area-level measurement of area-level economic deprivation and delay discounting, both of which found that higher levels of area-level deprivation were associated with steeper delay discounting, and one of which reported effect sizes in the moderate range (132, 133). We are not aware of previous studies examining the relationship between structural stressors, defined as stressors reflecting mutually reinforcing systems that place individuals from some groups at a disadvantage, and delay discounting.

The studies described above did not include neuroimaging. While many neuroimaging studies do not report effect sizes, moderate effect sizes have been found in the relationship between early childhood deprivation and brain volume in brain region including the IFG/insula and ACC (79, 81), and other studies have reported a relationship between neighborhood poverty and brain structure in dmPFC (83). Thus, given the exploratory nature of this study, and the paucity of previously reported effect sizes relating structural stressors, and delay discounting and neural correlates, we have used the existing literature to estimate that with *n* = 72 children we will be able to detect with 80% correlations at least as large as r = 0.35. This moderate effect size would provide evidence of a signal worth pursuing in the R33 phase.

All procedures described here are included in our study protocol, which was reviewed by the Columbia University IRB (protocol ID: AAAV4120; approved on 2/18/2025). Columbia University will act as the single IRB for the study, and both Columbia University and the University of Puerto Rico are recruitment sites for the study. For this pilot phase, we plan to invite 72 children (aged 11–14, 50% female, 50% from the South Bronx, 50% from Puerto Rico) to provide: home address history from the previous five years, substance use risk, BJW and magnetic resonance imaging (MRI) for brain structure and DD-related brain function.

Three inclusion criteria and two exclusion criteria will be ascertained before participants are enrolled in the study: Inclusion criterion 1. The child participant is a biological or non-biological child of a member of the original BYS sample. Inclusion criterion 2. The child participant is aged 11 to 14. Inclusion criterion 3. Parent/caregiver is between the ages of 18–65 in the continental US and 21–65 in Puerto Rico, where 21 is the age of majority. Exclusion criterion 1. The child participant has a major neurological disorder (e.g., seizure disorder) or cognitive impairment (e.g., moderate to severe Autism Spectrum Disorder, Intellectual Disability). Exclusion criterion 2. The child participant has MRI contraindications (e.g., irremovable metal on the body, pacemaker, braces, etc. For participants who meet inclusion and exclusion criteria and express interest in participating in the study, informed consent, assent and confidentiality are carefully explained to all eligible participants prior to asking parents to provide informed consent and children to provide informed assent.

We will be using a secure web-based central electronic data capture system the Research Electronic Data Capture (REDCap) for collection of interview data at both study sites. This electronic data capture system ensures the privacy and security of the data collected by meeting the Good Clinical Practice (GCP) guidelines and the following applicable Federal Regulations-Health Information Portability and Accountability Act (HIPAA). MRI data from both sites will be stored and processed on an enterprise-level web server via Xnat, an open-source imaging informatics software platform dedicated to imaging-based research.

Adolescent participants in NY and PR will be compensated $100 for participating in the MRI scan, will receive $10 as part of completing the DD task, and $100 for completing the interview, for a total of $210. Parents will also be compensated $100 for participating by providing interviews regarding their children.

### Statistical analyses

We will use generalized linear models (GLMs) using the SS composite variable as a predictor and brain structure in prefrontal regions of interest, DD-performance, neural correlates of DD performance and BJW as outcome variables. These GLMs will also control for family SUD factors, individual experiences of unfair treatment, age, sex, and the presence of a psychiatric diagnosis. Due to the importance of understanding SS alongside other negative exposures, our models will also include adverse childhood experiences, measured using a combination of parent and child report, gathered by interview.

Exploratory analyses will examine the role of each individual SS index on gray matter volume (e.g., the effect of area-level group differences in income alone) and will examine site differences (South Bronx vs. Puerto Rico) in the associations. Site differences may suggest site-specific approaches to prevention and intervention.

## Discussion

While SS, measured as area-level differences among groups, is recognized as a fundamental cause of disparities in health, it is currently unclear what are important mechanisms through which SS contributes to substance use risk in youth at high risk for substance use disorders. Characterizing how higher levels of SS could lead to greater risk via neurocognitive processes and elucidating specific brain-based mechanisms can improve understanding of neural correlates of SS and may provide compelling evidence to lawmakers to enact on structural change. The goal of an investigation with these objectives is to inform structural and family-level interventions aimed at reducing health disparities. While structural interventions are likely the best solution to eliminate structural disparities, elucidating mechanisms that can be modified can provide families with actionable information could allow families to make their own decisions and improve the wellbeing of their children. This is particularly important in situations and times periods when it is difficult to make structural changes to reduce health disparities.

Establishing the harms associated with SS and relevant mechanisms is necessary and important. However, also important is improving understanding of family-level protective and promotive factors. Thus, in the next phase of this study, we plan to examine factors that may prevent substance use in adolescence, as well as promotive factors. In addition, we plan to examine the moderating effect of family-level protective factors and explore how cultural factors can influence risk for SUD (134, 135) to determine if they can buffer the negative impact of SS on neurocognitive mechanisms of substance use risk. The long-term goal of this future work is to identify culturally relevant protective factors at the area-, family- and individual-level that can meaningfully inform lines of prevention and intervention.

Improved understanding of the relationship between SS indicators and mechanisms of substance use risk could shape future research examining the relationship between SS and substance use, as well as novel prevention and intervention efforts at the structural level, the desirable approach to addressing structural disparities and fundamental causes. Furthermore, results at the individual-level could suggest interventions such as episodic future thinking, which is aimed at helping individuals prioritize larger, later rewards over smaller, sooner rewards, could target reward-related decision making, as this intervention reduced DD and risky behaviors (136). Thus, the current study aims to examine the relationship between SS and mechanisms of substance use risk, in the service of informing future efforts at reducing the risk of adolescent substance use.
